# The Mediating Role of Overweight and Obesity in the Prospective Association between Overall Dietary Quality and Healthy Aging

**DOI:** 10.3390/nu10040515

**Published:** 2018-04-20

**Authors:** Karen E. Assmann, Indunil Ruhunuhewa, Moufidath Adjibade, Zhen Li, Raphaëlle Varraso, Serge Hercberg, Pilar Galan, Emmanuelle Kesse-Guyot

**Affiliations:** 1 Nutritional Epidemiology Research Team (EREN), Centre of Research in Epidemiology and Statistics Sorbonne Paris Cité, Inserm (U1153), Inra (U1125), Cnam, Paris 13 University, COMUE Sorbonne Paris Cité, 93017 Bobigny, France; indunil_ruhunuhewa@yahoo.com (I.R.); m.adjibade@eren.smbh.univ-paris13.fr (M.A.); s.hercberg@eren.smbh.univ-paris13.fr (S.H.); p.galan@uren.smbh.univ-paris13.fr (P.G.); e.kesse@eren.smbh.univ-paris13.fr (E.K.-G.); 2 INSERM, U1168, VIMA: Aging and Chronic Diseases, Epidemiological and Public Health Approaches, F-94807 Villejuif, France; lizhen1986@scu.edu.cn (Z.L.); raphaelle.varraso@inserm.fr (R.V.); 3 Université Versailles St-Quentin-en-Yvelines, UMR-S 1168, F-78180 Montigny le Bretonneux, France; 4 School of Public Health, Faculty of Medicine, Université Paris-Sud, 94276 Kremlin-Bicêtre, France; 5 Public Health Department, Avicenne Hospital, 93017 Bobigny, France

**Keywords:** aging, diet, nutrition, body mass index (BMI), overweight obesity, prevention

## Abstract

Background: Our objective was to quantify to what extent the association between adherence to the French nutritional recommendations at midlife, measured by the Programme National Nutrition Santé-Guideline Score (PNNS-GS), and healthy aging (HA) is mediated by body mass index (BMI) status. Methods: We analyzed data from 2249 participants of the French ‘Supplementation with Vitamins and Mineral Antioxidants’ (SU.VI.MAX-‘SUpplémentation en VItamines et Minéraux AntioXydants’) cohort. At baseline (1994–1995), data on BMI status (<25 vs. ≥25 and <30 vs. ≥30) and diet were collected. At follow-up (2007–2009), HA status (yes/no) was evaluated via a multidimensional concept focusing on chronic disease incidence, physical and cognitive functioning, mental and social health, pain, and perceived health. Relative risks (RR) were estimated by extensively adjusted robust-error-variance Poisson regression, and counterfactual-based mediation analysis was performed. Results: Our HA criteria were met by 39% of participants. We identified a positive direct relation of a greater adherence to the French nutritional recommendations, with the probability of HA (RR_Quartile 4 vs. quartile 1_ = 1.31 (95% confidence interval (CI) = 1.13, 1.53)), and an indirect relation mediated by BMI status (1.01 (95% CI: 1.01, 1.02)), accounting for 5% of the total relation. Conclusion: These results indicate that high dietary quality may contribute to the preservation of overall health during aging, partly via obesity prevention and partly via other mechanisms.

## 1. Introduction

The burden of age-related health decline and the dependence of the elderly population result in major societal implications [1]. To address these challenges, various public health promotion interventions for the aging population have been implemented [2]. Moreover, in geriatric research, multidimensional concepts of “successful aging” or “healthy aging” (HA) have emerged that have made it possible to consider different chronic diseases, as well as physical, cognitive, mental, and social health during aging in a combined, holistic manner [3]. The most well-known multidimensional concept of “successful aging” or HA is the framework proposed by Rowe and Kahn [4,5], which proposes three main dimensions of HA: avoiding disease and disability, maintaining high levels of physical and cognitive functioning, and having an active engagement with life [4]. The work of Rowe and Kahn notably underlines that there is a large heterogeneity in aging trajectories, and that premature overall health-decline is not inevitable, but is related to a diversity of parameters that include modifiable environmental factors [4]. In terms of the specific period during which such factors may have a particularly important role, midlife appears to be of particular interest. This period is considered a crucial time-window, since previous research has highlighted that age-related diseases and disabilities appear to be strongly related to risk factors present at midlife [6,7,8,9].

Several specific modifiable environmental factors have been proposed as determinants of overall health during aging, including physical activity [10], avoiding smoking [11], moderate alcohol consumption [11], and air pollution [12]. In addition, nutrition has been suggested to play a fundamental role in modulating healthy life expectancy [13]. Specifically, multiple observational studies have observed that individuals with a high overall dietary quality are more likely to age healthily [14,15,16,17,18,19,20].

In addition, much attention of both epidemiological [6,7,8,21,22,23,24] and mechanistic [25] research has been paid to the role of overweight and obesity for age-related chronic diseases and age-related functional decline. A main biological mechanism that appears to underlie the detrimental role of excess body fat, and especially central obesity, is adipose tissue inflammation. This process is characterized by an increased secretion of pro-inflammatory cytokines by adipocytes and the infiltration of immune cells (including macrophages and T-cells) into the adipose tissue. This adipose tissue inflammation appears to mediate insulin resistance [26], and has also been suggested to be involved in the etiology of cardiovascular disease [27], depression [28] and cognitive decline [29,30]. In line with this, various observational studies have revealed a detrimental role of an elevated body mass index (BMI) or an increased body fat percentage with respect to multidimensional concepts of HA [6,7,8,21,22,23,24,31], including a previous article based on data from the French ‘Supplementation with Vitamins and Mineral Antioxidants’ (SU.VI.MAX-‘SUpplémentation en VItamines et Minéraux AntioXydants’) study [31].

Obesity is a condition with multiple presumed determinants, including genetics, physical activity and sedentary time, unfavorable sleeping patterns, as well as diet [32]. Diet and physical activity/sedentary time are particularly important factors, since they are directly related to a disturbed energy balance, and thus a storage of excess energy as fat within the adipose tissue [33]. Multiple studies indicate that it is not only overall energy intake but also the quality of dietary patterns that is related to weight gain and obesity [34]. Given the importance of diet for avoiding overweight and obesity, and the key role of obesity as a determinant of health during aging [6,7,8,21,22,23,24], it is of interest to better characterize how the relationship between diet and overall HA is mediated by indicators related to body fatness.

Of note, the relationship between dietary quality and health is often modeled by adjusting for (amongst other things) BMI or BMI category. This approach makes it possible to isolate the “direct” role of diet for health outcomes, but it is questionable whether this is generally desirable or whether adjustment for mediating factors should be considered as overadjustment [35]. Most importantly, simply adjusting for BMI-related indicators does not make it possible to obtain specific insight into the direct and indirect association between diet and health outcomes. In the context of a potential mediation by BMI status, the “direct” association can be characterized as the part of the relationship that is mediated by mechanisms that are independent of overweight and obesity. On the other hand, the “indirect” association can be conceptualized as the part of the relationship that is mediated by mechanisms related to excess body fat.

To the best of our knowledge, no previous study has specifically focused on the mediating role of BMI status in the relationship between overall dietary quality and multidimensional healthy aging. Thus, our objective was to quantify the extent to which the association between adherence to nutritional recommendations at midlife and later healthy aging is mediated through BMI status, while distinguishing 3 categories: (a) underweight/normal weight (BMI < 25 kg/m^2^), (b) overweight (BMI ≥ 25 and <30 kg/m^2^), and (c) obesity (BMI ≥ 30 kg/m^2^). Adherence to the French official nutritional guidelines for the general population was measured by the Programme National Nutrition Santé-Guidelines Score (PNNS-GS).

## 2. Materials and Methods

### 2.1. Study Population

The randomized, double-blinded placebo-controlled trial ‘Supplementation with Vitamins and Mineral Antioxidants’ (SU.VI.MAX-‘SUpplémentation en VItamines et Minéraux AntioXydants’) was conducted in 1994–2002 to test the effect of a daily supplementation with antioxidant vitamins and minerals at nutritional doses on the incidence of cancer, cardiovascular disease and overall mortality [36]. This trial included 12,741 men and women. Next, a post-supplementation observational study ‘SU.VI.MAX 2’ was conducted in 2007–2009 and 6850 subjects were recruited on a voluntary basis.

The SU.VI.MAX and SU.VI.MAX 2 studies were conducted according to the Declaration of Helsinki, and were approved by the ethics committee for studies with human subjects of Paris-Cochin Hospital (CCPPRB number 706 and number 2364, respectively) and the “Commission Nationale de l’Informatique et des Libertés” (CNIL number 334641 and number 907094, respectively). All participants provided written informed consent. The SU.VI.MAX trial was registered at www.clinicaltrials.gov/ct2/show/NCT00272428.

**Figure 1:**
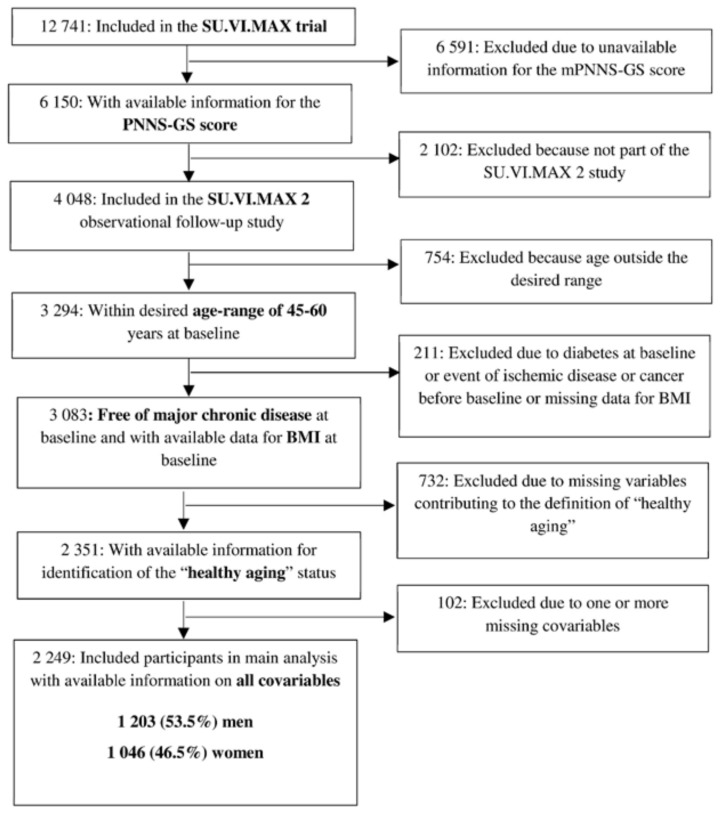
Sample selection of participants from the ‘Supplementation with Vitamins and Mineral Antioxidants’ (SU.VI.MAX-‘SUpplémentation en VItamines et Minéraux AntioXydants’) study, France, 1994–2009; body mass index (BMI); Programme National Nutrition Santé-Guideline Score (PNNS-GS).

The eligibility criteria for the present analysis were: being a participant of the SU.VI.MAX 2 study with available and accurate (at least three 24 h dietary records) information on adherence to the French dietary guidelines (*N* = 4048), an age of 45–60 years at recruitment (1994–1995), the absence of chronic diseases (cancer, cardiovascular disease or diabetes) at recruitment in 1994–1995 (*N* = 3083), and the absence of missing values for baseline BMI and for variables necessary to determine HA status in 2007–2009 (*N* = 2351). Individuals with missing data for baseline covariables—such as sociodemographic factors (gender, age, educational level, occupation, and family situation), smoking status and number of 24 h records—were also excluded, leaving a final sample of 2249 individuals (Figure 1).

### 2.2. Data Collection

#### 2.2.1. Timeline of Data Collection

All participants included in our analyses were aged 45–60 years at inclusion in the initial SU.VI.MAX study (1994–1995). Moreover, they were free of cancer, cardiovascular disease and diabetes at recruitment (1994–1995). The dietary data used for the current analyses were collected in 1994–1996, and the anthropometric data used were collected during a clinical examination in 1995–1996. Participants were followed for a median of 13 y, until the SU.VI.MAX 2 observational follow-up point in 2007–2009.

Of note, from inclusion (1994–1995) until 2002, participants either received a placebo or a daily dose of antioxidant nutrients for approximately 8 years (1994–2002), according to the design of the initial SU.VI.MAX trial. In the present analyses, this antioxidant supplementation was only considered as an adjustment variable.

#### 2.2.2. Healthy Aging (HA) Status (2007–2009)

The main outcome measure of our study was HA status (yes/no), defined on the basis of a multidimensional concept that included the following eight criteria [37]: absence of incident major chronic disease (cancer, cardiovascular disease or type II diabetes) during follow-up, good cognitive functioning, good physical functioning, absence of limitations in instrumental activities of daily living, absence of depressive symptomatology, absence of health-related limitations in social life, good overall self-perceived health, and absence of function-limiting pain. Participants were characterized as aging healthily when all eight HA components were met. HA status was determined between 2007 and 2009. More details on this HA definition and on each specific component are provided in Table 1. Additional information has been published elsewhere [37].

#### 2.2.3. Baseline Dietary Data (1994–1996)

Participants of the SU.VI.MAX trial were asked to complete one 24 h dietary record every 2 months. Accordingly, computerized questionnaires and an instruction manual including validated photographs of more than 250 foods were provided. Seven possible portion sizes were available to choose from [46]. A detailed description of dietary data treatment in the SU.VI.MAX study has been published previously [47]. Briefly, dietary records reporting energy intakes of <100 kcal/d or >6000 kcal/d were considered as invalid. To further account for energy under-reporting, men reporting < 800 kcal/d and women reporting <500 kcal/d across ≥1/3 of valid records were excluded. Baseline questionnaires were used to collect data on alcohol and seafood consumption, as these food groups tend to be consumed less frequently than others. Alcohol consumption was estimated by a short, validated, semi-quantitative dietary questionnaire [48]. The average number of dietary records available per participant was 10.2 (interquartile range: 8–13). All eligible 24 h records collected during the first 2 years following inclusion were used to compute food and nutrient intakes. Details on the computation of the PNNS-GS have been published previously [49]. Briefly, the maximum number of points is 15, and the score includes 13 components: eight concern food serving recommendations (fruit and vegetables, starchy foods, whole grain food, dairy products, meat poultry seafood and eggs, seafood, vegetable fat and non-alcoholic beverages), 4 concern moderation (added fat, salt, sweetened foods, alcohol), and 1 concerns physical activity. Points are deducted for overconsumption of salt and sweets and for elevated energy intakes.

#### 2.2.4. Anthropometric Data (1995–1996)

Weight was measured to the nearest 0.5 kg using an electronic scale (Seca, Hamburg, Germany), with participants wearing indoor clothing and no shoes. Height was measured to the nearest 0.5 cm with a wall-mounted stadiometer under the same conditions. A “BMI status”—variable of 3 categories was created, which distinguished: (a) underweight/normal weight (BMI < 25 kg/m^2^), (b) overweight without obesity (BMI ≥ 25 and <30 kg/m^2^), and (c) obesity (BMI ≥ 30 kg/m^2^).

#### 2.2.5. Baseline Covariates (1994–1996)

Self-administered questionnaires were completed at recruitment (1994–1996), including information on gender, date of birth, educational level (primary/secondary/university or equivalent), occupation (homemaker/manual worker/office employee/intellectual profession or managerial staff), family situation (living alone/cohabiting), smoking status (non-smoker/former smoker/current smoker), number of 24 h records, and antioxidant supplementation (yes/no).

### 2.3. Statistical Analysis

#### 2.3.1. Descriptive Statistics and Results on the Simple Association between Dietary Quality and HA

Baseline characteristics of participants were compared according to HA status (yes/no), using Mann-Whitney U tests and Chi^2^-tests.

In order to describe the relationship of the PNNS-GS with HA, we used robust-error-variance Poisson regression models, which make it possible to estimate relative risks (RRs) and 95% confidence intervals in the presence of a frequent binary outcome [50].

Analyses on the relationship between the PNNS-GS and HA were presented for the overall PNNS-GS score and for modified scores, obtained by sequentially removing each of the 13 components from the PNNS-GS one by one, with further adjustment for the removed component. The aim of these analyses was to test whether any of the score components explained a major part of the association between the overall PNNS-GS and HA, or whether the association was due to a combination of all components.

Models were adjusted for age, gender, smoking status, educational level, occupational status, living situation, antioxidant supplementation, total energy intake and number of 24 h records.

#### 2.3.2. Main Analysis: Mediating Role of BMI Status in the Association between Diet Quality and HA

The PNNS-GS score (modeled as quartiles) was the observed exposure of interest (*A*), BMI status was the mediator (*M*) and HA the outcome (*Y*). Mediation analysis was conducted according to a counterfactual-based method proposed by Lange et al., which has similarities with marginal structural models, and allows to estimate natural direct and indirect effects [51]. Briefly, this method includes the following steps:

(1) Creation of a new dataset by repeating each observation 4 times, including a new variable A*, which corresponded to each of the 4 PNNS-GS quartiles. This new variable A* was equal to *A* (the genuinely observed PNNS-GS quartile) for one line per subject and different from A for the 3 other lines;

(2) Two multivariable (i.e., confounder-adjusted) logistic regression models were applied to the new dataset to estimate the association between PNNS-GS quartiles (*A*/A*) and BMI status (*M*), first using the original variable *A*, and then the new variable A*;

(3) ‘Individual stabilized weights’ were calculated using the predicted probabilities obtained from these two logistic regressions, as follows:

(1) $$W_{i}^{C}=P(M=M_{i}|A=A_{i}^{*},\;C=C_{i})/P(M=M_{i}|A=A_{i},\;C=C_{i})$$

where *i* denotes the individual, *A* the observed exposure, A* the created exposure, *M* the mediator, and *C* denotes the different potential confounders;

(4) A weighted Poisson regression model was applied to estimate the association between PNNS-GS quartiles (*A* and A*) and HA. This Poisson model and the above-mentioned logistic regression model were all adjusted for age, gender, smoking status, educational level, occupational status, living situation, antioxidant supplementation, total energy intake and number of 24 h records. The RR associated with the variable *A* was interpreted as the direct effect of PNNS-GS quartiles with respect to HA, and the RR associated with the variable A* as the indirect effect. The total effect was calculated as the product of the natural direct effect and the natural indirect effect. For the total effect, 95% confidence intervals were derived from a bootstrap sample distribution (*N* = 1000). The proportion mediated by BMI status was computed using the following formula:

(2) $$\left[{\mathrm{RR}}^{\mathrm{NDE}}\left({\mathrm{RR}}^{\mathrm{NIE}}-1\right)]\;÷\;[{\mathrm{RR}}^{\mathrm{NDE}}\;\times {\mathrm{RR}}^{\mathrm{NIE}}-1\right]\;\times \;100\%$$

where NDE is natural direct effect and NIE is natural indirect effect.

The mediation analysis was conducted under the working hypothesis that the following underlying conditions were satisfied [51]: (1) no unmeasured confounders for the associations between: (a) PNNS-GS quartiles and HA, (b) BMI status and HA, (c) PNNS-GS quartiles and BMI status; and (2) no association between confounders of the relation between BMI status and HA with PNNS-GS quartiles.

All analyses were performed using 9.4 version of the SAS software (SAS Institute Inc., Cary, NC, USA).

## 3. Results

### 3.1. Descriptive Statistics

On average, participants were aged 51.9 (SD = 4.5) years at baseline, and the average follow-up duration was 13.5 (SD = 4.3) years. The prevalences of overweight (excluding obesity) and obesity were 30.8% and 5.8%, respectively.

A quite large proportion of participants were defined as “aging healthily”; 42% and of men and 36% of women (39% in total). Table 2 shows characteristics of participants defined as “aging healthily”, as compared to their counterparts who did not meet ≥ 1 of the criteria of our HA definition. As expected, these “healthy agers” were younger, more educated, and were part of more advantaged occupational categories. In addition, a larger proportion of men than of women in our sample were defined as “healthy agers”, and “healthy agers” were more often cohabiting than living alone. Finally, participants that showed good overall health during aging had a higher physical activity level, showed a higher level of adherence to the French nutritional recommendations, had a lower BMI, and a higher fiber intake.

### 3.2. Relationship between Dietary Quality and Overall Healthy Aging (HA)

Results concerning the associations between overall dietary quality (measured by the PNNS-GS) and HA status are displayed in Table 3. There was an overall positive association between higher scores on the PNNS-GS and the probability to age healthily, and this remained true even when removing different components of this score (representing different dimensions of dietary quality). Of note, the strength of the association between the dietary scores and HA was quite similar when comparing the original PNNS-GS and versions where specific components had been removed (relative risks ranged from 1.04 to 1.07, and all tested associations were statistically significant).

### 3.3. The Mediating Role of Body Mass Index (BMI) Status

Table 4 shows the results of our analysis that aimed to characterize the mediating role of BMI status in the association between dietary quality and HA. This analysis indicates a quite small but statistically significant mediating role of BMI status; for the comparison of the highest vs. lowest PNNS-GS quartile, the estimated percentage of the association with HA mediated by BMI status was 5%. This corresponds to a ‘natural indirect effect’ relative risk estimate of 1.31 (95% confidence interval = 1.13, 1.53), as compared to a ‘natural direct effect’ relative risk estimate of 1.01 (95% confidence interval = 1.01, 1.02).

## 4. Discussion

The findings of the present investigation suggest that greater adherence to the French nutritional recommendations at midlife was longitudinally associated with HA, through a direct effect and to a lesser extent an effect mediated by BMI status. The role of corpulence was small but not negligible, indicating that high dietary quality may in part contribute to the preservation of good overall health via the prevention of obesity.

In analyses on the link between adherence to the French national nutrition recommendations and HA, all nutritional scores (the global PNNS-GS and the different modified scores created by removing each component one by one) were associated with HA, arguing for a role of overall adherence to the French nutritional recommendations-rather than a specific role of one or several particular guidelines.

A number of studies have investigated the relationship between the overall diet, using holistic approaches, and multidimensional concepts of HA [14,15,16,17,18,19,20]. Although indicators of dietary quality were quite heterogeneous across studies, the observed findings overall argue for an important role of nutrition for preserving good overall health during aging.

Midlife BMI and/or overweight and obesity have been identified as major determinants of HA measured by multidimensional concepts [7,22,23,31], amongst others in a previous investigation of data from the SU.VI.MAX cohort [31]. In that previous investigation, obesity was associated with a significantly reduced probability to age healthily, while overweight was only linked to a decreased probability of HA when associated with impaired metabolic health [31]. Hence, it should be noted that while the current study used a 3-class indicator of BMI status (i.e., underweight/normal weight/overweight/obesity), it can be assumed that the mediation effect observed is mainly attributable to the avoidance of obesity and less to the avoidance of overweight.

On the other hand, in the British Whitehall II cohort, both overweight and obesity (as well as a large waist circumference), at midlife were related to a lowered probability of “successful aging”, defined as the absence of coronary heart disease, stroke, cancer and diabetes; good cardiovascular, metabolic, respiratory, physical and cognitive functioning; and the absence of mental health problems [22].

To the best of our knowledge, our study is the first to specifically and formally investigate the mediating effect of BMI status in the relationship between diet and healthy aging. While certain investigations on the link between diet and HA have not taken BMI into account [17,18], an investigation of data from the Nurses’ Health study has presented analyses adjusted for BMI [19]. However, since this was done in a non-sequential manner (i.e., no otherwise fully-adjusted model excluding BMI was shown in addition to the fully-adjusted model including BMI), it is not possible to specifically characterize the mediating role of BMI based on these results. On the other hand, a study based on the Melbourne Collaborative cohort has presented such sequentially adjusted models, and the conclusions that can be drawn from these data are concordant with our results [18]. Indeed, after additional adjustment for BMI and waist-to-hip ratio, an only partial attenuation of the association between an a posteriori dietary pattern high in meat and fatty foods and HA was observed (OR_4th quartile compared to the 1st_ of 0.69 (95%CI = 0.55–0.86) versus 0.78 (95%CI = 0.62–0.98)). However, sequential adjustment for BMI does not allow to assess a mediating effect of corpulence in an exact, quantitative manner.

In our study, the proportion of the overall association that was estimated to be mediated by BMI status was quite low (5%). This suggests that healthy aging may be promoted by a healthy diet through specific nutritional effects in the “direct” pathway, and to a lesser extent through mechanisms related to the prevention of overweight and obesity. Scientific literature examining the role of bioactive nutritional compounds and overall dietary patterns on different components of the healthy aging is plentiful [52,53,54,55]. Potential mechanistic pathways include low-grade chronic inflammation [55], oxidative stress [56], DNA methylation and other epigenetic mechanisms [53], vascular damage [57], and dysbiosis of the gut microbiota [52]. In particular, low-grade chronic inflammation is affected by corpulence (since adipose tissue inflammation has been suggested to mediate comorbidities of obesity such as the metabolic syndrome and cardiovascular disease [58]), but appears to also be directly related to the consumption of specific food groups, nutrients, and overall dietary patterns [59,60].

Some limitations of the present study should be noted, which include residual confounding due to potential confounders such as life events and psychological factors that were not taken in to account. Furthermore, participants of the SU.VI.MAX 2 prospective study were originally volunteers who took part in the SU.VI.MAX randomized trial. This may have resulted in a lack of representativeness of the general population, and particularly in a lower prevalence of obesity. Hence, the proportion of the association mediated by obesity may be greater in the general population than in our study sample. It should also be noted that it is difficult in observational research to model the natural course over which diet presumably influences BMI status, which may then be involved in the etiology of age-related diseases and functional decline. Here, we considered both BMI status and dietary intakes assessed at baseline, but it can be assumed that dietary habits at earlier ages, including childhood, may have influenced BMI status at baseline. This may be one explanation for the rather small mediating effect of BMI status observed in the current study.

In addition, part of the data that was used to define HA status was exclusively collected during the SU.VI.MAX 2 observational study. The presence and consideration of such baseline health data would have increased the robustness of our results to potential reverse causation bias.

The main strengths of this study are of the prospective study design and the large sample size. Moreover, using a multidimensional HA concept helped to better capture the broad variety of aspects related to physical and mental wellness in a global manner. Another positive feature of the present study was the use of counterfactual-based mediation analysis, which permitted the formal investigation of the mediating role of BMI in the association between diet and HA.

In conclusion, this prospective investigation of data from a large French cohort provides novel evidence that high adherence to the French nutritional recommendations at midlife contributes to the preservation of health during aging, beyond potential impacts on obesity prevention. To our knowledge, the present study is the first to formally characterize the mediating role of BMI status in the relationship between dietary quality and a multidimensional concept of healthy aging. From a public health point of view, both dietary advice and weight management may help to promote a better overall health status during aging. However, since only a small part of the association between dietary quality at midlife and HA appeared to be mediated by BMI status, it can be hypothesized that increasing overall dietary quality may be beneficial even beyond targeting weight loss or weight stabilization. Further prospective observational studies and intervention trials are required to confirm these findings.

## Figures and Tables

**Table 1:** Definition of Healthy Aging status in the trial ‘Supplementation with Vitamins and Mineral Antioxidants’ (SU.VI.MAX-‘SUpplémentation en VItamines et Minéraux AntioXydants’) study.

Dimension ^1^	Definition
Good physical functioning (yes/no)	Short Physical Performance Battery (SPPB) score ≥11/12 The SPPB includes 3 tests (administered by trained physicians): Repeated chair stands, balance testing, gait speed testing [38]. Higher scores on the SPPB indicate better performance.
Good cognitive functioning (yes/no)	Mini Mental State Examination (MMSE) score ≥27/30 and *rappel indicé—48 items* (RI-48) score ≥19/48 and Delis-Kaplan Trail-making test (DK-TMT) scaled score ≥5.5 The MMSE [39] tests global cognitive functioning, the RI-48 tests verbal episodic memory [40], and the DK-TMT tests mental flexibility [41,42]. Higher scores on each test indicate better performances. All three tests were administered by trained neuropsychologists or geriatric physicians.
No limitations in IADL (yes/no)	<1 limitation on the Lawton Scale of Instrumental Activites of Daily Living (IADL) The Lawton IADL scale [43] is a self-administered questionnaire (including, amongst others, questions on the ability to travel, go shopping, and do housekeeping). Having more limitations on the scale indicates lower independence in daily life.
No depressive symptoms (yes/no)	Center for Epidemiological Studies Depression Scale (CES-D) score <16/60 The CES-D [44] is a self-administered questionnaire developed for the evaluation of depressive symptoms in the general population, within epidemiological studies. Higher scores indicate more depressive symptoms.
No health-related limitations in social life (yes/no)	No/only slight and only infrequent (perceived) interference of health problems with social life Data were collected via the Medical Outcome Study Short Form-36 (SF-36) questionnaire [45], a very widely used, self-administered questionnaire designed to measure vitality, physical functioning, bodily pain, general health perceptions, physical role functioning, emotional role functioning, social role functioning, and mental health
Good overall self-perceived health (yes/no)	To meet this criterion, participants had to declare that their health was generally “good” to “excellent” Participants’ responses were collected via the SF-36 questionnaire [45].
No function-limiting pain (yes/no)	To meet this criterion, participants had to report having experienced no more than “mild” physical pain during the previous month or that such pain had only limited or no impact on their daily activities Participants’ responses were collected via the SF-36 questionnaire [45].
No incident major chronic disease (yes/no)	No incident cancer, cardiovascular disease, or diabetes during follow-up Events of cancer and of cardiovascular disease were recorded during follow-up and validated by an independent external committee of medical doctors. Cancer was defined as cancer of any kind, except for basal cell carcinoma. Cardiovascular disease was defined as codes I20–I25, I63, I65, I66, I70, I71, and I74 from the 10th International World Health Organization Classification of Diseases). Incident diabetes was defined as having a fasting blood glucose value ≥ 1.26 g/L, anti-diabetic medication use, or self-reported diabetes at the end of follow-up.
Overall healthy aging	Participants meeting all of the above criteria were considered to be aging healthily, while participants for which at least one criterion was = “no” were not considered to be aging healthily → Thus, a binary indicator of overall healthy aging was obtained (yes/no).

^1^ All criteria were assessed at follow-up (2007–2009), apart from events of major chronic disease, for which incidence during follow-up (1994–2009) was assessed.

**Table 2:** Baseline characteristics of the study population according to healthy aging status, SU.VI.MAX 2 study (France), 2007−2009, *N* = 2249.

	Healthy Aging Status		
Sample Characteristics ^a^	Number	Yes	*P*-Value ^b^
Number (%)	1368 (60.8)	881 (39.2)	
Age, y, mean (SD)	52.3 (4.6)	51.3 (4.3)	<0.0001
Gender (male), %	51.3	56.9	0.01
Region of residence, %			0.19
Larger Paris-region (“Ile de France“)	22.2	20.8	
North-east and north-west of France	15.8	13.1	
West, mid-west, and “Rhone Alpes”- regions	45.3	48.6	
South-west and Mediterranean regions	16.7	17.6	
Educational level, %			<0.0001
Primary education only	23.6	16.6	
Secondary education	40.4	38.5	
University level	36.0	44.9	
Occupational status, %			<0.0001
Homemaker	8.0	6.2	
Manual worker	6.8	4.1	
Office employee	57.2	52.0	
Intellectual profession ^c^	28.0	37.7	
Smoking status, %			0.10
Non-smoker	50.1	51.8	
Former smoker	38.4	39.5	
Current smoker	11.5	8.7	
Family situation			0.01
Living alone	14.2	10.7	
Married/cohabiting	85.8	89.3	
Physical activity level, %			<0.0001
Irregular or none	25.9	18.5	
<1 h/day	30.0	29.8	
≥1 h/day	44.1	51.7	
Antioxidant supplementation (yes), %	51.5	54.7	0.14
Alcohol consumption, g/day, mean (SD)	16.8 (17.4)	17.0 (17.2)	0.40
Number of 24h records, mean (SD)	10.1 (3.2)	10.4 (2.9)	0.04
PNNS-GS (points), mean (SD)	7.7 (1.9)	8.0 (1.9)	0.001
Body mass index (kg/m^2^), mean (SD)	24.5 (3.5)	24.0 (2.9)	0.01
Body mass index status, %			0.0002
BMI < 25 kg/m^2^	61.5	66.3	
BMI ≥ 25 and <30 kg/m^2^	31.1	30.3	
BMI ≥ 30 kg/m^2^	7.4	3.4	
Total energy intake (kcal/d), mean (SD)	2194.5 (629.0)	2221.7 (595.7)	0.22
Protein (% energy/d), mean (SD)	16.7 (2.6)	16.5 (2.5)	0.09
CarbohyDrates (% energy/d), mean (SD)	39.6 (6.7)	39.9 (6.3)	0.55
Total fat (% energy/d), mean (SD)	37.6 (5.1)	37.6 (4.7)	0.88
SFA (% energy/d), mean (SD)	15.4 (2.7)	15.5 (2.5)	0.71
MUFA (% energy/d), mean (SD)	14.2 (2.2)	14.2 (2.1)	0.85
PUFA (% energy/d), mean (SD)	5.7 (1.5)	5.7 (1.4)	0.93
Fiber intake (g/d), mean (SD)	20.1 (7.3)	20.6 (7.1)	0.04
Sodium intake (mg/d), mean (SD)	3613.6 (1231.7)	3623.2 (1188.1)	0.72

Abbreviation: SFA: Saturated fatty acids; MUFA: Monounsaturated fatty acids; PUFA: Polyunsaturated fatty acids ^a^ Values are mean (standard deviation, SD) or % as appropriate. ^b^ *P*-values are based on Mann-Whitney U tests (continuous variables) and Chi-square tests (categorical variables). ^c^ Or managerial staff; Programme National Nutrition Santé-Guideline Score (PNNS-GS).

**Table 3:** Relative risk estimates (95% confidence interval (CI)) for the association between nutritional requirement (PNNS-GS) components and healthy aging status, SU.VI.MAX 2 study (France), 2007–2009, *N* = 2249 ^a,b.^

Nutritional Score	RR (95% CI)	*p*-Value
PNNS-GS	1.06 (1.03, 1.09)	0.0001
PNNS-GS without Physical activity component	1.04 (1.01, 1.08)	0.0109
PNNS-GS without Seafood component	1.06 (1.02, 1.09)	0.0009
PNNS-GS without Fruits and vegetables component	1.06 (1.03, 1.10)	0.0006
PNNS-GS without Whole grain food component	1.06 (1.03, 1.10)	0.0001
PNNS-GS without Total added fats component	1.06 (1.03, 1.09)	0.0002
PNNS-GS without Salt component	1.06 (1.03, 1.09)	0.0002
PNNS-GS without Vegetable added fats component	1.07 (1.04, 1.10)	<0001
PNNS-GS without Alcohol component	1.06 (1.03, 1.09)	0.0002
PNNS-GS without Meat and poultry, seafood and eggs component	1.06 (1.03, 1.09)	0.0002
PNNS-GS without Milk and dairy products component	1.06 (1.03, 1.09)	0.0001
PNNS-GS without Sweetened foods component ^c^	1.06 (1.03, 1.09)	0.0002
PNNS-GS without Bread, cereals, potatoes and legumes	1.06 (1.03, 1.09)	0.0001
PNNS-GS without Beverages component ^d^	1.06 (1.03, 1.09)	0.0002

^a^ Robust-error-variance Poisson regression models were used to model healthy aging status. ^b^ Models were adjusted for age, gender, smoking status, educational level, occupational status, living situation, antioxidant supplementation, total energy intake, number of 24 h records and the removed component, except for the overall PNNS-GS. ^c^ Points for the sweetened foods- component were calculated based on added sugar from sweetened foods (including food items such as sweet cakes, cookies, and chocolate), as a % of daily overall energy intake (% EI/d): (a) ≥17.5% EI/d → −0.5 points; (b) 17.5–12.5% EI/d → 0 points; (c) <12.5% EI/d →1 point. ^d^ Points for beverages- component were calculated as follows: (a) <1l water and >250 ml soda/d → 0 points; (b) ≥1l water and >250 ml soda/d → 0.5 points; (c) <1l water and ≤250 ml soda/d → 0.75 points; (d) ≥1l water and ≤250 ml soda/d → 1 point; Programme National Nutrition Santé-Guideline Score (PNN-GS); Relative risk (RR).

**Table 4:** Relative risk estimates for the direct and the indirect effect ^a^ for the mediating role of BMI status on the association between the PNNS - Guidelines Score and healthy aging, SU.VI.MAX 2 study (France), 2007–2009, *N* = 2249.

	PNNS-GS Quartiles RR (95% CI) ^b^				
	Q1 <6.55	Q2 (6.55–7.80)	Q3 (7.80–9.05)	Q4 >9.05	P_trend_
Direct effect ^a^	1.00 (ref)	1.08 (0.92, 1.26)	1.20 (1.03, 1.40)	1.31 (1.13, 1.53)	0.0002
Indirect effect ^c^	1.00 (ref)	1.01 (1.00, 1.01)	1.01 (1.00, 1.01)	1.01 (1.01, 1.02)	0.0003
Total effect ^d^	1.00 (ref)	1.09 (0.92, 1.26)	1.21 (1.03, 1.41)	1.33 (1.13, 1.55)	
Mediation (%) ^e^		7%	5%	5%	

^a^ Based on the method of mediation analysis proposed by Lange et al. (2012). Adjusted for age, gender, smoking status, educational level, occupational status, living situation, antioxidant supplementation, total energy intake and number of 24 h records. ^b^ The natural direct and indirect effects were obtained from the weighted mediation model of robust-error-variance Poisson regression. ^c^ Corresponds to the value of the exposure relative to the indirect path. ^d^ Confidence intervals for the total effect were assigned by bootstrapping. ^e^ The proportion mediated was computed as $\left({\mathrm{OR}}^{\mathrm{NDE}}\left({\mathrm{OR}}^{\mathrm{NIE}}-1\right))\;÷\;({\mathrm{OR}}^{\mathrm{NDE}}\;\times {\mathrm{OR}}^{\mathrm{NIE}}-1\right)\;\times \;100\%$. NDE-natural direct effect; NIE-natural indirect effect; Programme National Nutrition Santé-Guideline Score (PNNS-GS).
